# Metal Nanostructures for Environmental Pollutant Detection Based on Fluorescence

**DOI:** 10.3390/nano11020276

**Published:** 2021-01-21

**Authors:** Luca Burratti, Erica Ciotta, Fabio De Matteis, Paolo Prosposito

**Affiliations:** 1 Department of Industrial Engineering and INSTM, University of Rome Tor Vergata, Via del Politecnico 1, 00133 Rome, Italy; luca.burratti@uniroma2.it (L.B.); demattei@roma2.infn.it (F.D.M.); 2 Institute for Microelectronics and Microsystems (IMM) CNR Tor Vergata, Via del Fosso del Cavaliere 100, 00133 Rome, Italy; erica.ciotta@artov.imm.cnr.it

**Keywords:** noble metal nanomaterials, engineered nanomaterials, fluorometric sensors, water pollutants, heavy metals detection, pesticides detection, water monitoring

## Abstract

Heavy metal ions and pesticides are extremely dangerous for human health and environment and an accurate detection is an essential step to monitor their levels in water. The standard and most used methods for detecting these pollutants are sophisticated and expensive analytical techniques. However, recent technological advancements have allowed the development of alternative techniques based on optical properties of noble metal nanomaterials, which provide many advantages such as ultrasensitive detection, fast turnover, simple protocols, in situ sampling, on-site capability and reduced cost. This paper provides a review of the most common photo-physical effects impact on the fluorescence of metal nanomaterials and how these processes can be exploited for the detection of pollutant species. The final aim is to provide readers with an updated guide on fluorescent metallic nano-systems used as optical sensors of heavy metal ions and pesticides in water.

## 1. Introduction

Industrial human activities and technological advancements are increasingly burdening the environment with the release of large quantities of hazardous waste, such as heavy metals, metalloids and organic pollutants. This pollution has inflicted serious damage on ecosystems [1]. The build-up of heavy metal content in soils and waters continues to induce serious problems for global health, as these pollutants cannot be degraded into non-toxic forms but they accumulate in the ecosystem [2,3] also reaching the food chain [4].

The use of pesticides in commercial agriculture has led to an increase in farm productivity [5]. Sure, they are designed to be toxic to parasites but unfortunately, by their very nature, they pose risks to humans, wildlife and the environment.

Table 1 shows the guidelines for the maximum tolerated concentration of the main pesticides in drinking water for some countries, while Table 2 shows those for metal ions in water. In both cases, there is no homogeneity of values among countries. It seems noteworthy that the European Union (EU) has the strictest limits for both types of pollutants.

In order to monitor the amount of heavy metal ions and pesticides accumulated in nature, a wide range of sample types, such as soil, water, air, food, beverage, plant and animal-derived products, needs to be tested routinely. The most widely used analytical methods to detect the presence of contaminants in real samples are: atomic absorption spectroscopy (AAS) [6,7], atomic emission spectroscopy (AES) [8,9], mass spectroscopy (MS) [10,11] and chromatography methods [12,13]. All of them have excellent sensitivity and reproducibility, however, their protocols for sample preparation are time-consuming [14,15,16,17,18] and they require very expensive and sophisticated apparatuses, handled by well-trained personnel. For these reasons, it is worth exploring possible easier and lower-cost alternatives. In this context, optical detection techniques based on nanomaterials with peculiar optical properties seem a very promising cheaper and easier method. Indeed, they have faster detection times, simple protocols and they allow in situ sampling and portability. In addition, the optical methods exploit the ability of specific materials to change their optical properties such as reflectance/transmittance, absorbance and fluorescence in presence of contaminants and these changes can be investigated by simple apparatuses such as spectrophotometers or fluorimeters.

**Figure 1:**
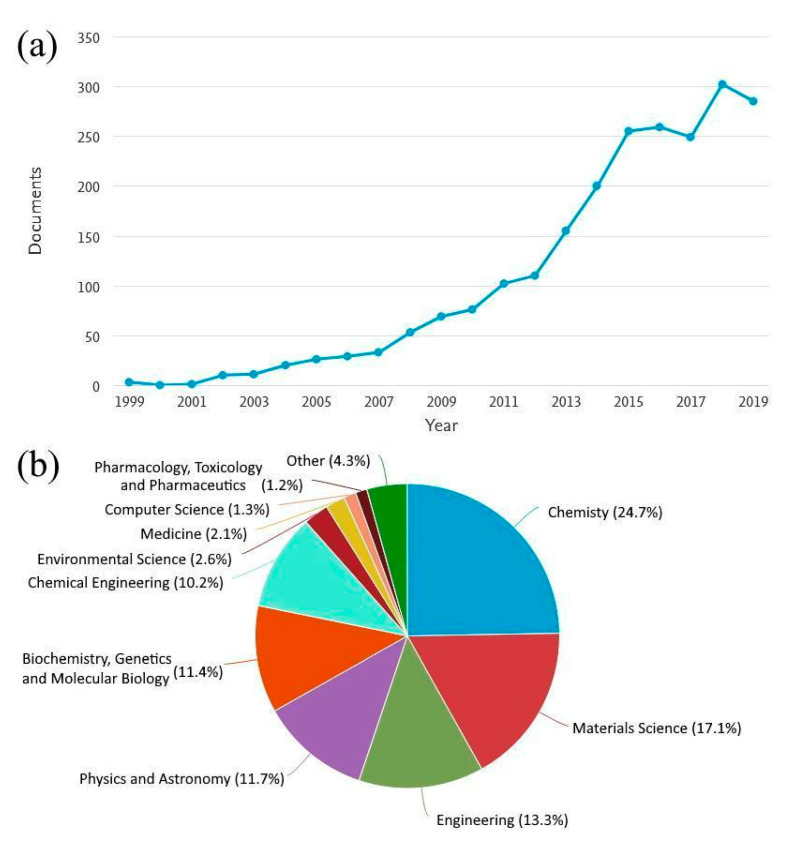
Analysis on the published manuscripts in the time range from 1999 to 2019: documents by year (**a**) and by subject area (**b**), respectively. Source: www.scopus.com.

An analysis of publication data from the Scopus website highlights that the scientific interest on the fluorescent nanomaterials for sensor applications increased in the last twenty years (1999–2019), as reported in Figure 1a. The number of publications (containing keywords: *nanoparticles*, *fluorescent* and *sensor*) jumped from 3 in 1999 to 285 in 2019. While the Figure 1b shows the published manuscripts in the same time range divided by subject areas, the main fields are chemistry and materials science, with 24.7% and 17.1% of the total of manuscripts, respectively. The number of papers in the environmental science area passed from 0% of publication in the 1999 to 8% in the 2019, showing the increased interest of this research field.

Nanosensors are sensors used to detect the presence of chemical species on the nanoscale. Fluorescent nanomaterials such as inorganic quantum dots (QDs) [19,20,21] perovskite quantum dots (PQDs) [22,23,24], graphene quantum dots (GQDs) [25,26,27,28] are employed as nanosensors for environmental monitoring. Moreover, other nanostructures are used as fluorescent probes for pollutant detection such as noble metal nanoclusters (MNCs) [29,30,31,32] or functionalized nanoparticles [33,34,35]. In the case of noble metal nanoclusters, the fluorescence is an intrinsic property of the material itself and it is connected with the small dimensions that induce a quantization of the energy levels; in other cases and with specific functionalization, luminescent species or molecules are bond directly to the nanoparticles that do not show intrinsic fluorescence [36,37,38]. When such nano-systems interact with contaminants, the fluorescence properties such as intensity, peak position and band shape undergo a modification. Clearly, the interaction mechanisms depend on the nature of the analyte and on the specific nanosensor considered. In general, interactions occur directly on the surface of the metal nano-systems. Alternatively, the molecules that surround the nanostructures mediate these interactions by their functional groups.

This review aims to describe the most common photo-physical effects involved in the fluorescence of the noble metal nanostructures and their modifications induced by the presence of pollutant species, such as heavy metal ions and pesticides, which are the widest diffused and harmful water and environmental contaminants. Photoinduced electron transfer (PET) and photoinduced charge transfer (PCT) processes, fluorescence resonance energy transfer (FRET) mechanism, aggregation-induced emission (AIE) effect and the inner-filter effect (IFE) are the kind of photo-physical effects commonly exploited in nanosensing. Moreover, we discuss a number of recent and promising systems used for detection of heavy metal ions and pesticides, highlighting their performances in terms of linear range, selectivity and limit of detection (LOD).

**Table 1:** Comparison between different guideline values for pesticides in drinking water [39]. For EU the Drinking Water Directive (DWD) sets a concentration limit of 0.1 μg/L for individual pesticides and of 0.5 μg/L for the total sum of pesticides [40]. Values are expressed in μg/L, N. a. = not approved.

Pesticide	* WHO	** USA	New Zeeland	Australia	Canada	*** EU
Alachlor	20	2	20	-	-	N. a.
Aldicarb	10	7	10	1	9	N. a.
Aldicarb sulfone	-	7	-	-	-	N. a.
Aldicarb sulfoxide	-	7	-	-	-	N. a.
Aldrin/diedrin	0.03	-	0.03	0.01	0.7	N. a.
Atrazine	2	3	2	0.5	5	N. a.
Bentazone	300	-	4	2	-	0.1
Carbaryl	-	-	-	5	90	N. a.
Carbofuran	7	40	8	5	90	N. a.
Chlordane	0.2	2	0.2	0.01	-	N. a.
Cyanazine	0.6		0.7	-	10	N. a.
2,4-D	30	70	40	0.1	100	0.1
DDT	2	-	-	-	-	N. a.
Diazinon	-	-	10	1	20	0.1
1,2-Dibromo-3-chloropropane	1	0.2	1	-	-	N. a.
Diquat	10	20	10	0.5	70	0.1
EDB	0.4–15	0.05	-	1	-	N. a.
Fenoprop(2,4,5-TP)	9	50	10	-	-	N. a.
Glyphosate	-	700	-	10	280	0.1
Heptachlor	-	0.4	-	-	-	0.1
Heptachlor epoxide	-	0.2	-	-	-	N. a.
Hexachlorobenzene	1	1	1	-	-	0.1
Lindane	2	0.2	2	0.05	-	0.1
Malathion	-	-	-	50	190	0.1
Methoxychlor	20	40	20	0.2	900	0.1
Pentachlorophenol	9	1	10	0.01	60	0.1
Picloram	-	500	20	-	190	0.1
Propazine	-	-	70	0.5	-	0.1
Simazine	2	4	2	0.5	10	0.1
2,4,5-T	9	-	10	0.05	-	N. a.
Trifluralin	20	-	30	0.1	45	0.1

* WHO = World Health Organization; ** USA = United State of America; *** EU = Europe.

**Table 2:** European limit values [41] for heavy metal concentration in comparison with WHO [42] and EPA* [43] values (all expressed in mg/L).

Metal ions	EU	WHO	EPA
Iron (Fe)	0.2	0.3	0.3
Manganese (Mn)	0.05	0.05	0.05
Copper (Cu)	1	1	1.3
Zinc (Zn)	0.1	5	5
Silver (Ag)	0.01	-	-
Arsenic (As)	0.05	0.01	0.01
Cadmium (Cd)	0.005	0.005	0.005
Chromium (Cr)	0.05	0.05	0.1
Mercury (Hg)	0.001	0.001	0.002
Nickel (Ni)	0.05	-	0.1
Lead (Pb)	0.05	0.015	0.005

* EPA = Environmental Protection Agency.

## 2. Physical Aspects behind Fluorescence Response to Environmental Pollutants

The nature of the metal nanomaterials and the pollutant species is the key element to determine the type of interaction and the effect on the fluorescent emission band. In this section, the most important interaction mechanisms between the probe system and the pollutants will be discussed.

### 2.1. Photoinduced Electron Transfer and Photoinduced Charge Transfer Processes

**Scheme 1:**
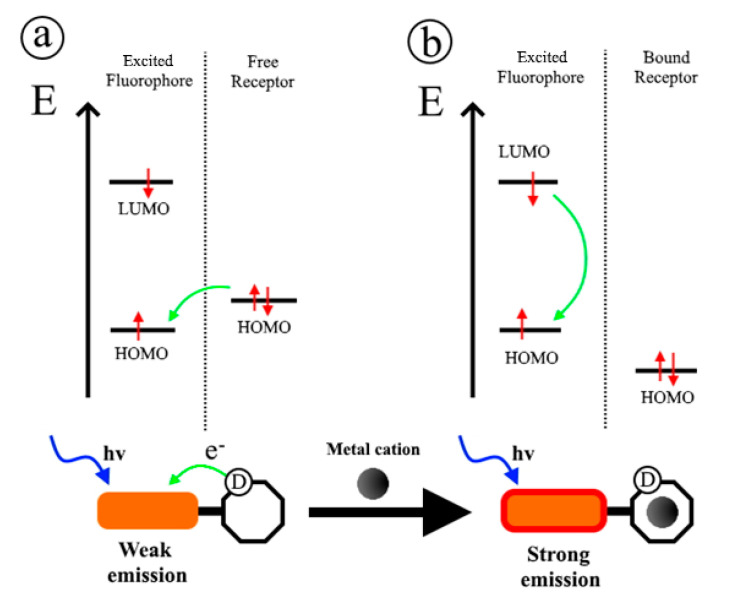
Energy levels and electrons involved in the transitions before (**a**) and after (**b**) the interaction with the metal cation, in the photoinduced electron transfer (PET) mechanism.

**Scheme 2:**
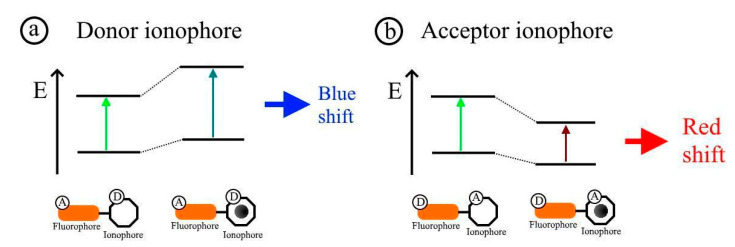
Energy levels involved in the transitions before and after the interaction with metal cations in the donor ionophore case (**a**) and in the acceptor ionophore case (**b**), for the photoinduced charge transfer (PCT) mechanism.

Photoinduced electron transfer (PET) sensors are based on molecules having a fluorophore moiety, responsible for the light emission and an ionophore part, involved in the interaction with the heavy metal ion (see Scheme 1a). When the system is free from contaminant, after optical excitation, the emission intensity is weak, due to an electron transfer from the ionophore energy level to the excited fluorophore level that inhibits the radiative transition between the two states of the fluorophore moiety. During the contamination with metal cations, the probability of radiative transition is enhanced [44,45], caused by the inhibition of the electron transfer from the ionophore to the fluorophore, since the binding with the cation stabilizes the energy level of the ionophore (see Scheme 1b). Other mechanisms causing an enhancement or quenching of the fluorescence intensity are chelation-enhanced fluorescence (CHEF) and chelating quenching fluorescence (CHQF) [46,47,48]. Moreover, heavy metal ions can induce aggregation of the emitting units and, if the aggregation is strong enough, complete vanishing of the emitted light may occur. These interaction mechanisms explain the signal increasing/decreasing of some systems in literature [47,49,50,51,52,53,54]. More complex is the case of photoinduced charge transfer (PCT) based sensors. In this case, a different behavior occurs if the ionophore moiety is an electron acceptor or electron donor species. When a fluorophore contains an electron-donating ionophore conjugated to an electron-accepting group, an intramolecular charge transfer from the donor to the acceptor occurs upon excitation by light. When an electron donor group within the fluorophore interacts with a cation, a blue shift of the absorption and emission spectra is expected. In fact, the metal cation reduces the electron-donating character of this group, strongly destabilizing the excited state with respect to the ground state. A schematic representation is reported in the Scheme 2a.

Conversely, a cation interacting with an acceptor group enhances the electron-attraction character of this group; consequently, the cation stabilizes more efficiently the excited state rather than the ground state, causing a red shift of the absorption and emission spectra (see Scheme 2b) [44].

### 2.2. Fluorescence Resonance Energy Transfer Mechanism

**Scheme 3:**
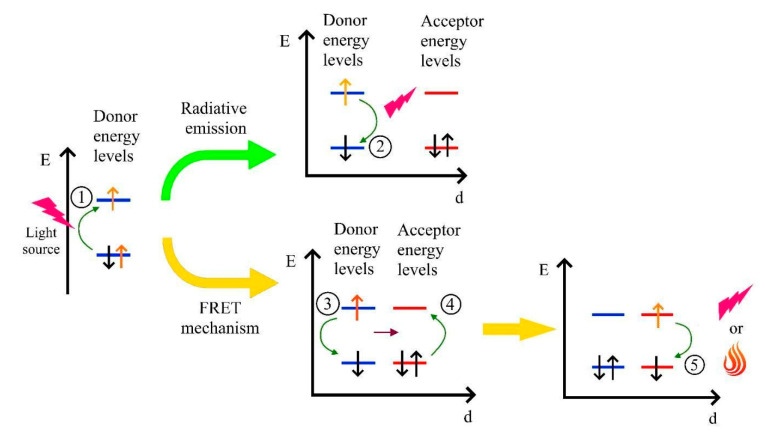
Schematic representation of processes involved between two fluorophores, a donor and an acceptor in the fluorescence resonance energy transfer (FRET) mechanism.

Fluorescence (or Föster) resonance energy transfer (FRET) is a non-radiative process, which occurs among fluorescent molecules. This phenomenon is based on long-range dipole-dipole interaction between a donor fluorophore (D) in its excited state and a proximal ground state of an acceptor fluorophore (A) [55]. Two main conditions must be satisfied, in order for the FRET process to occur. First, the donor and acceptor must be located in a close proximity up to a maximum distance of 10 nm. Second, the overlapping area of the emission spectrum of donor and the absorption spectrum of acceptor must be large enough. On the contrary, if these conditions are not fulfilled, FRET does not occur and in the case of donor excitation, donor relaxes to its ground state by radiative emission [56,57]. The relaxation mechanisms that can occur once the donor compound is in its excited state, is shown in the Scheme 3, where the distance *d* between donor and acceptor dipoles is represented in abscissa. An electromagnetic wave of appropriate wavelength excites the donor (process ①). Two relaxation mechanisms for the donor can occur following excitation. The first one is through radiative emission (process ②, if the distance *d* > 10 nm and/or overlapping of bands is not satisfied). The second one is through FRET, where the de-excitation process ③ occurs and the excess of energy (represented by horizontal violet arrow) is transferred to the acceptor moiety, exciting the acceptor itself (process ④). Finally, the acceptor loses the excess of energy returning to the fundamental state by radiative or non-radiative emission (process ⑤) [55,58].

When a metal nanoparticle replaces one of the two fluorophores involved in the FRET phenomenon, the energy transfer no longer occurs between the two dipoles but rather among the excited dipole and the conduction band electrons of the metal nanoparticle. This phenomenon is known as nanometal surface energy transfer (NSET). Furthermore, the NSET mechanism can occur at a longer distance than the FRET, up to about 20 nm [59]. The energy levels representation is still valid also in the case of NSET, considering the due replacements.

This phenomenon involving metal nanoparticles functionalized to a fluorophore can be exploited to detect environmental pollutions [60,61,62,63,64,65]. It is possible to identify two sensor classes. In the first type, the presence of the pollutant promotes NSET phenomenon, the donor fluorophores transfer the energy to the acceptor nanoparticles and thus a quenching of photoluminescence (PL) emission occurs. In the second type, a fluorescence “turn-on” effect occurs, for the contaminant inhibits the energy transfer from donor to acceptor (often due to an aggregation of the particles) and the fluorophores far from the metal structures can emit radiatively.

### 2.3. Aggregation-Induced Emission Effect

The aggregation-induced emission (AIE) effect is a photo-physical property of those molecules with an electronic structure extensively conjugated [66]. The AIE effect is the ability of these species to increase their PL emission when they are in an aggregate status. This macroscopic effect is mainly caused by the restriction of intramolecular rotation (RIR) in the aggregates, since the intramolecular rotation converts photon energy into heat and it deactivates the excited states via non-radiative processes. In the aggregates, the intramolecular rotation is restricted and the non-radiative relaxation channel is reduced [66]. Metal nanomaterials sensors based on the AIE effect have been applied to water monitoring in several cases, where the presence of water pollutants induces the aggregation of the fluorescent probes and subsequently, the increase of the emission [67,68,69].

### 2.4. Inner-Filter Effect

The inner-filter effect (IFE) is another photo-physical phenomenon, which can be exploited in the field of environmental monitoring. When a fluorescent compound is in a concentrated solution and excited by light, the fluorescence originates only from the solution surface, inasmuch fluorophore molecules absorb all the excitation energy in the first layers of the solution. The penetration power of the light decreases, increasing the distance from the surface, thus the light is absorbed and cannot excite the innermost molecules. Therefore, there is a non-linear relationship between the observed fluorescence intensity and the concentration of emitters [70,71].

A sensor based on IFE is composed of two optical units, that is, an absorber (metal NPs) and an emitter (fluorophore). In order to synthesize a good IFE sensor, the absorption spectrum of the absorber should possess a sufficient overlap region with the excitation and/or emission spectrum of the emitter, as result the fluorescence emission is tuned by the absorber and the fluorescence should not be quenched by the absorber [72].

**Scheme 4:**
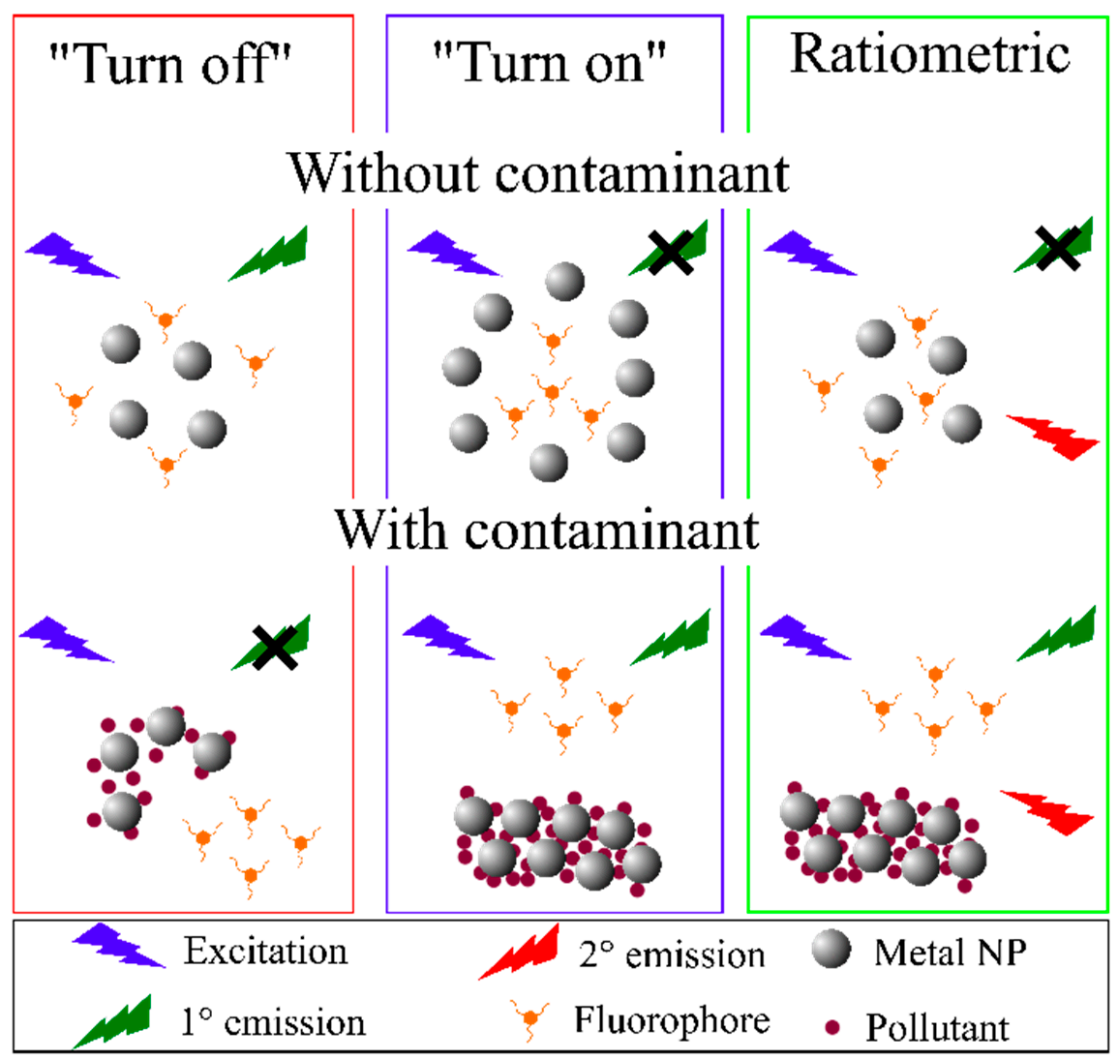
Strategies of inner-filter effect (IFE) based sensors.

The Scheme 4 shows the three strategies for the design of the IFE based fluorescent sensing systems. The first strategy consists in the fluorescence “turn-off,” that is, the absorption of the metal NPs is enhanced with the increase of analyte concentration giving rise to a decrease of the fluorescence of the system due to IFE. The IFE-based sensors in a “turn-on” mode represents the second strategy, where the NPs turns off the fluorescence first and then the analyte restores the emission intensity mostly by the aggregation of metal NPs [73]. The third strategy is ratiometric fluorescence assay in which the analyte concentrations are determined by measuring the ratios of the emission at two wavelengths (represented in the Scheme 4 by green and red lightnings). Here the pollutant affects one of the two emission and as a result the emission color changes [74,75].

## 3. Recent Developments on the Heavy Metal Ions Detection

### 3.1. Silver

Silver is used in many different fields, mostly to exploit its antibacterial and antibiotic activity and for this reason it is present in high quantities in the environment. At certain levels, it can destroy the activity of enzymes and for this reason, it is important to have systems able to detect it in water even at low concentration. For example, fluorescence polarization sensors are used for Ag(I) detection in aqueous solution. Thiol-DNA-functionalized gold nanoparticles (AuNPs) [33,76] were recently used because Ag(I) ions interact with a cytosine–cytosine (C–C) mismatch in DNA duplexes to form stable metal-mediated cytosine–Ag(I)–cytosine (C–Ag(I)–C) base pairs. The formation of this complex causes a variation in the molecular volume and fluorescence polarization signal. After testing the system with 12 different cations, the authors found a LOD of 9.5 nM for silver ions in water and good results with interfering ions were obtained.

A common Ag(I) detection technique in water was obtained using fluorescent copper nanoparticles synthesized by using glucose (Glc) as a reducing agent and fluorescent probe (Glc-CuNPs). Silver ions interact with Glc-CuNPs quenching the fluorescence signal [77]. 18 different metal ions were tested in this system and a LOD of 100 µmol/L was attained.

Another system based on fluorescence quenching to detect Ag(I) ions in water consists of fluorescent carbon-doped silicon nanoparticles (SiNPs) [78]. The fluorescence signal of SiNPs was quenched in the case of Ag(I) ions and Hg(II) ions, with detection limits of 0.457 μM and 2.676 μM, respectively. The system was tested in the presence of 15 different metal ions but only in the presence of Ag(I) and Hg(II) ions there was a quenching effect. Furthermore, the interference between different metal ions was tested but there were no influences in the fluorescent intensity of the system.

A different sensing mechanism is based on fluorescence enhancement in the presence of Ag(I) ions. Gold nanoclusters (AuNCs) covered with glutathione were synthesized to detect silver ions in water. In this case, the enhancement of the luminescence was due to aggregation-induced emission of the functionalized gold nanoclusters [79]. The system was tested with 13 different metal ions but a luminescence enhancement with red-shift of the emission peak was measured only when Ag(I) ions were present in water. The authors suggested that Ag(I) influenced the ligand-to metal charge transfer (LMCT) or ligand-to metal–metal charge transfer (LMMCT) of AuNCs and they found a limit of detection of 0.2 nM.

### 3.2. Chromium

Chromium, in aqueous solutions, can be found in two different oxidation states, Cr(III) and Cr(VI). The former has low toxicity, while the latter is a human carcinogen. Fluorescent silicon nanoparticles were reported to be a good sensor for Cr(VI) since the signal is quenched in presence of this ion through the inner filter effect [80] with a LOD of 28 nM. The system was tested with 17 different ions and the interference of these ions with Cr(VI) showed no influence. After the interaction with Cr(VI) the fluorescence of the system can be recovered by adding H_2_S due to the redox reaction between Cr(VI) and H_2_S, so this on-off-on sensor is also able to detect H_2_S in water.

**Figure 2:**
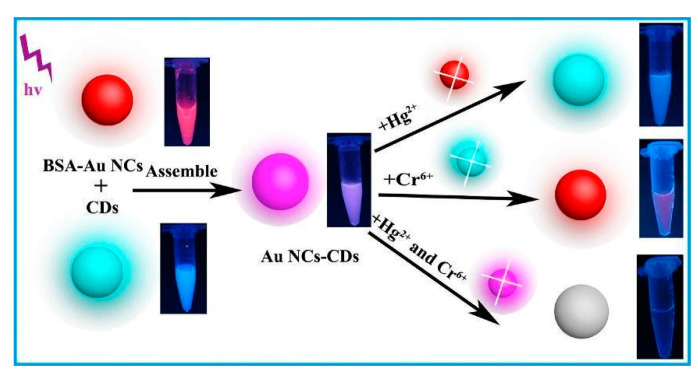
The schematic illustration of the synthetic procedure of BSA-AuNCs-CDs and detection of metal ions by BSA-AuNCs-CDs. (Reprinted from reference [81] with permission from Elsevier).

In another work, bovine serum albumin (BSA)-AuNCs and carbon dots CDs (BSA-AuNCs-CDs) systems were synthesized to detect Cr(VI) (LOD 5.34 nM) and also Hg(II) (LOD 1.85 nM) [81]. In this work, 13 different metal ions were tested. As shown in the schematic illustration of Figure 2, the red fluorescence of BSA-AuNCs and the blue fluorescence of CDs are combined giving a pink fluorescence signal. In the presence of mercury or chromium ions there was a change in the fluorescence emission color which allows to detect the specific ion.

The quenching of CDs fluorescence upon addition of Cr(VI) may be attributed to the inner filter effect and the detection of Hg(II) is based on the high-affinity metallophilic Hg(II)–Au(I) interactions, which quenches the fluorescence of AuNCs. An interesting system based on glutathione-stabilized gold nanoclusters (GSH-AuNCs) was tested with 10 different cations and shows a selective detection of both Cr(III) and Cr(VI) in environmental water samples by fluorescence quenching depending on the pH [82]. At pH 6.5, a selective quenching effect was found for Cr(III) with a LOD of 2.5 µg/L. In these conditions, Cr(III) ions can form complexes with glutathione (AuNCs-GSH-Cr(III)-GSH-AuNCs) resulting in the aggregation of AuNCs. Moreover, the system presented a selective quenching effect (LOD of 0.5 µg/L) for pH values between 3.5–1, caused by the redox interaction between Cr(VI) and GSH that determines an instability of AuNCs with aggregation.

### 3.3. Cadmium

Cadmium is a dangerous heavy metal ion for human health because it can bind with different amino acids that play important metabolic functions and inhibit them.

**Figure 3:**
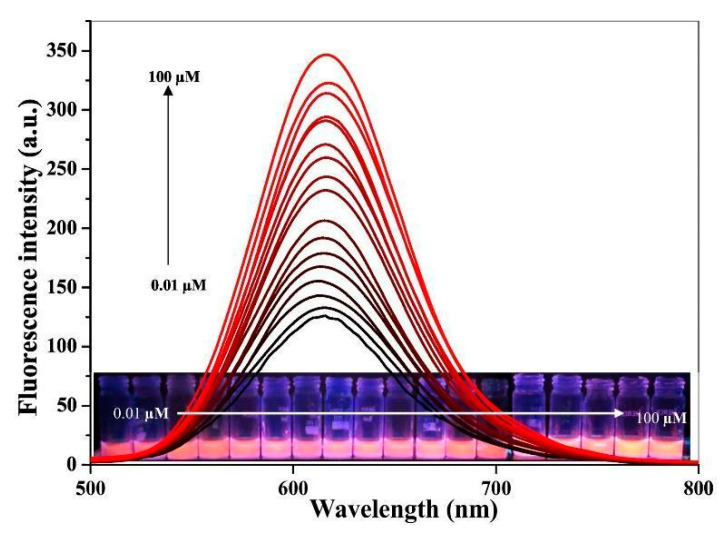
Fluorescence emission response of CE/11-MUA-AuNCs upon addition of Cd(II) in the range 0.01–100 μM. (Adapted from reference [67] with permission from Elsevier).

There are different ways to use metal nanoparticles for Cd(II) detection. A common detection mechanism for Cd(II) ions is based on fluorescent enhancement mechanisms as shown in Figure 3. For example, orange-emitting fluorescence AuNCs with methionine as a stabilizer, was synthesized to detect Cd(II) ions in water by a fluorescent enhancement mechanism with a LOD of 12.25 nM. In this case 10 cations and 11 anions were tested but only the presence of Cd(II) in solution causes the AuNCs aggregation due to the link between Cd(II) and Au via chelating bonds with a carboxyl group or an amino group in methionine adsorbed on the Au nanoclusters [83].

Orange red-emitting AuNCs derived from *Curcuma longa root* extract (CE) and 11- mercaptoundecanoic acid (11-MUA) (CE/11-MUA-AuNCs) as reducing and capping ligands were tested with 12 different metal ions and was used for the detection of Cd(II), Zn(II) (with an increasing of fluorescence intensity) and Cu(II) (with a decreasing of fluorescence intensity) in aqueous solution. In this case the lowest LOD was 0.012 µM for Cd(II) ions, for the other two ions the LOD was for Zn(II) 0.016 µM and for Cu(II) 0.026 μM, respectively [67]. In addition, in this case there is no interference between different ions. The fluorescence enhancement was due to CHEF and to AIE processes.

An alternative process which produces an increase of fluorescence signal in the presence of Cd(II) ions is PET as reported for AuNCs-BSA (Bovine serum albumin) systems [84]. In this case, 9 different cations were tested but only cadmium ions can be detected in the range 5–165 ng/mL.

On the other side, quenching mechanisms are also used for cadmium detection. One example is represented by carbon quantum dots/gold nanoclusters (CQDs/AuNCs) in which 12 cations were tested. These nanohybrids were used to detect Cd(II) ions in water by fluorescent quenching mechanism with a LOD of 0.105 µM [74].

Another system, tested with 11 different cations, is based on OMRTH (octamethoxy resorcin arene tetrahydrazide)-AgNPs [85] in which quenching mechanism in the presence of Cd(II) ions was attributed to nanoparticle aggregation induced by the complexation between OMRTH and Cd(II). A LOD of 10^−8^ M was attained in this case.

### 3.4. Copper

Copper is an important element for human beings and its deficiency can result in anemia, neutropenia and bone abnormalities. However, an excessive exposure of copper has deleterious effects on human’s health and causes diseases such as vomiting, cramps, kidney and liver damage or even death for very high ingestion.

Sensitive sensors for its detection are very important and many studies were recently reported. For instance, L-tyrosine capped silver nanoparticles have been synthesized with a new procedure that allows to bind a fluorescent chromophore (the un-oxidized tyrosine), directly on the nanoparticles surface. The use of silver colloids, offers a great advantage since Ag exhibits a very high efficiency of plasmon excitation, resulting in increased sensitivity. The selectivity was not investigated and the same system give response also for Co(II) ions. A sensitivity of about 40 ppb has been reached which is comparable to that of traditional methods based on UV-Vis spectroscopy [86].

An interesting alternative for the synthesis of fluorescent silver nanoparticles based on a green protein R-phycoerythrin (R-PE) extracted from marine Porphyra yezoensis used both as stabilizer and reducer has been proposed [87]. Such a system presented a good response to Cu(II) ions based on the aggregation of the R-PE-AgNPs and a consequent fluorescence quenching. The system was tested with 15 different heavy metal ions showing a very high selectivity for copper ions. The system was also tested in tap and lake water samples giving very good results with low interference between the presence of different ions and with a LOD of 0.0190 μM.

**Figure 4:**
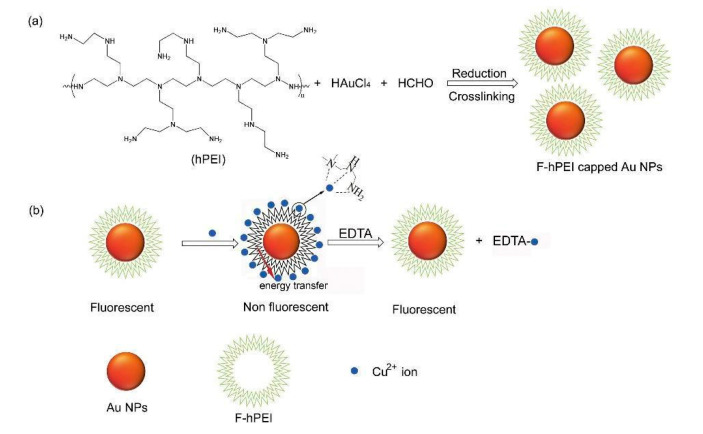
Proposed (**a**) reaction mechanism and (**b**) fluorescence quenched and recovered mechanism for the Cu (II) ion sensor based on F-hPEI capped AuNPs. (Reprinted from reference [88] with permission of Elsevier).

Recently, a fluorescent and colorimetric sensor for Cu(II) ion based on formaldehyde modified hyperbranched polyethylenimine capped gold nanoparticles (F-hPEI AuNPs) has been proposed by Wang et al. [88]. The possible fluorescence intensity quenching mechanism for the Cu(II) sensor is based on the energy transfer from F-hPEI to Cu(II) ions chelated with the amine groups. The mechanism is shown in Figure 4, where the recovering process based on ethylenediaminetetraacetic acid disodium salt (EDTA) is also reported. The recovery mechanism is explained considering that the EDTA binding with Cu(II) ions leads to a separation of the Cu(II) ions from the amine groups. Interference study was carried on showing that the presence of other contaminants does not affect the copper ions sensitivity. Such system was tested also in real water samples showing good and precise determination of Cu(II) ions.

Another method improving the selectivity of gold nanoclusters toward copper and mercury ions was developed [89]. In this case, gold nanoclusters are stabilized with bovine serum albumin and responded to Cu(II) and Hg(II) ions through fluorescence quenching. EDTA and sodium borohydride have the function of “masking” reagents, where EDTA complexed with Cu(II) and borohydride reduced Hg(II) into Hg(0). Both these reactions inhibited the ions interaction with the Au nanoclusters and eliminated quenching effect, allowing the detection of the other ions. The selectivity using different cations was also tested and the response was positive only for copper and mercury ions. LOD for Cu(II) was estimated to be about 300 nM and 8 nM for Hg(II).

### 3.5. Mercury

Mercury is one of the most dangerous heavy metal ions for humans and the environment. The most common metal nanoparticles (or nanoclusters) used for the detection of this heavy metal ion are those based on silver and gold.

Fluorescent silver nanoclusters (AgNCs) synthesized with denatured bovine serum albumin (dBSA) as stabilizing agents, were used to detect Hg(II) ions with a LOD of 10 nM exploiting fluorescence quenching effect. In this case 10 different cations were tested. The effect was due to the specific 5d^10^(Hg(II))-4d^10^(Ag(I)) metallophilic interaction between Hg(II) and Ag(I) onto the dBSA coated Ag clusters [90].

In another work [91], AgNCs were synthesized with dihydrolipoic acid as a stabilizing agent. The authors explain the quenching effect in the presence of Hg(II) ions in two different ways. A quenching effect occurs due to aggregation between Hg(II) ions and AgNCs but the fluorescence quenching can also be due to the donation of electrons from the AgNCs to the Hg(II) ions, which causes a partial Ag oxidation and an amalgam formation and as consequence AgNCs goes to a nonfluorescent form. In this case, the LOD was about 10^−10^ M. This effect does not occur in the presence of the other 12 different metal ions that were tested and no interference occurs between the ions.

Fluorescent gold nanoclusters (AuNCs) in aqueous media can be used to detect Hg(II) ions thanks to the high-affinity metallophilic Hg(II)–Au(I) interactions, which quenches the fluorescence of AuNCs [92]; in this case 15 different cations were tested and the quenching effect occurred in the presence of Hg(II) with a LOD of 0.5 nM. This NCs can be functionalized in different ways, for example luminescent AuNCs were synthesized by using keratin as template followed by a simple modification with silver(I) ions (AuNCs-Ag@Keratin). The authors found that in the presence of Hg(II) ions (LOD of 2.31 nM) a quenching effect takes place due to the oxidation of Ag(0) to Ag(I) by reduction process of Hg(II) ions, subsequently was re-oxidized by sulfhydryl groups in keratin [93]. The system was tested with 11 different cations and no effect of interference has been detected.

In another example, fluorescent AuNPs were functionalized with 11-mercaptoundecanoic acid (11-MUA) (11-MUA-AuNPs) to detect Hg(II) ions based on aggregation-induced quenching process [34]. In this case, the LOD of the system was 5 nM. The aggregation was due to the interaction between the carboxylate groups present on the surfaces of the 11-MUA-AuNPs and the Hg(II) ions by a chelation process. The system with 18 different cations was tested and in addition to Hg(II), also in the presence of Pb(II) and Cd(II) there was a variation of fluorescence intensity.

In some cases, non-fluorescent nanoparticles can be used as fluorescence systems after a functionalization with a specific dye to detect mercury ions by a quenching process. An example is represented by gold nanoparticles functionalized with a mercury specific DNA (MSD), that is rich in thymine (T) and can form the configuration T–Hg(II)–T in the presence of Hg(II) ions. The MSD was labelled on one side with a dye, that could be a fluorescein (FAM) [65,94] or a QD [64] and on the other side with a thiol group linked to an AuNPs. In the presence of Hg(II) ions, there was the formation of the T–Hg(II)–T configuration, that causes a change in the MSD conformation from random coil to hairpin-link structure. As a result, dye and AuNPs will be in close contact and the fluorescence resonance energy transfer process between the energy donor (dye) and the energy acceptor was exploited.

Another important method to detect Hg(II) is the “turn-on” fluorescence method. In this case, the system was tested in presence of 8 metal ions. In the presence of Hg(II) there was a fluorescence enhancement, as in the case of gold nanoparticles (AuNPs)/rhodamine B (RB)/hexanedithiol (HDT) nanocomposites system [95]. According to the authors Hg(II) ions can bind with AuNPs or thiol terminated AuNPs/RB/HDT causing the displacement of the RB molecules into the solution, in this case the LOD was 0.5 ppb.

**Figure 5:**
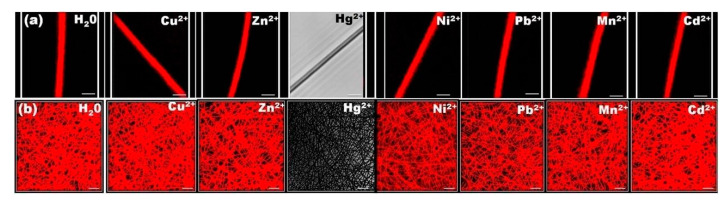
Selective sensing performance of AuNCs-PCL single nanofiber and AuNCs-PCL nanofibers matrix. Confocal laser scanning microscopy image presents the fluorescence response of AuNCs-PCL single nanofiber (**a**) and AuNCs-PCL nanofibers matrix (**b**) to various metal ions (indicated in each image) at a concentration of 10 ppm. Scale bar figure (**a**) 2 μm and (**b**) 5 μm. (Adapted from reference [96]).

As shown in Figure 5, fluorescent nanoparticles can be used in membranes and not only in water solution. In this case, 7 different ions were used to test the system. Polycaprolactone (PCL) nanofibers decorated with AuNCs (AuNCs-PLC) were used for the real-time detection of Hg(II) ions in water [96]. The AuNCs can bind the nanofiber surface thanks to the presence of carbonyl groups in the PCL and the carboxylic acid groups capped on the surface of the AuNCs. This system can detect Hg(II) ions with a LOD of 50 ppt thanks to the enhanced interaction between gold and mercury, as a consequence of a rapid adsorption and a formation of Au-Hg amalgam.

### 3.6. Manganese

Manganese ions are important for human health and are essential for biological systems, however high Mn(II) ions accumulation can cause serious problems such as Parkinson disease and neuronal defects in biological systems. For these reasons, it is important to have sensors sensitive to this specific contaminant.

A system based on water-soluble silicon nanoparticles (SiNPs) having a strong blue luminescence has been developed by Meng et al. [97]. The system presents a quenching of the fluorescence as a function of the Mn(II) ions due to the SiNPs aggregation (“turn-off”). A linear behavior with a detection limit of 1.1 μM was found. Moreover, the fluorescence quenching can be restored in the presence of EDTA because the EDTA can grab Mn(II) from the aggregated SiNPs-Mn(II) system owing to its strong chelating ability (“turn-on”). The system shows a high selectivity for manganese ions and in addition the interference study shows a low interference only with Cu(II) and Fe(III) ions. Moreover, the system was validated also in real water samples showing its feasibility for Mn(II) ions detection in practical applications.

Rich-thymine (T) single-strand DNAs have been employed as a template to synthesize water-soluble copper nanoclusters (CuNCs@T) for the first time [98]. The as-prepared CuNCs@T exhibited yellow photoluminescence. Such CuNCs@T can used as practical sensor for “turn-on” luminescence with red-shifted emission detection for Mn(II) in water solution with a high selectivity (between 14 different metal cations), a LOD of 10 μM and a linear response as a function of the ions concentration in the range from 100 μM to 250 μM. The system was also tested in real water samples with good sensitivity results.

### 3.7. Lead

Lead ions are between the most toxic heavy metal ions. They have severe risks and causes adverse health effects on different organs such as liver, kidney, brain and central nervous system, high blood pressure and even delayed physical and mental development in children. The possibility to check its presence in drinking water even in very small amounts is therefore highly desirable.

A “turn-on” fluorescent biosensor based on silver nanoclusters using an aptamer DNA as stabilizer was recently reported [99]. The fluorescence intensity of DNA-AgNCs enhances significantly in the presence of Pb(II) ions, due to the special interaction between Pb(II) and its aptamer DNA. Pb(II) ions can be detected up to 3.0 nM within a good linear range from 5 to 50 nM. The selectivity of the system was tested for different metal ions showing a very high selectivity to lead ions. Moreover, tests in real water samples demonstrates the reliability of this sensor.

**Figure 6:**
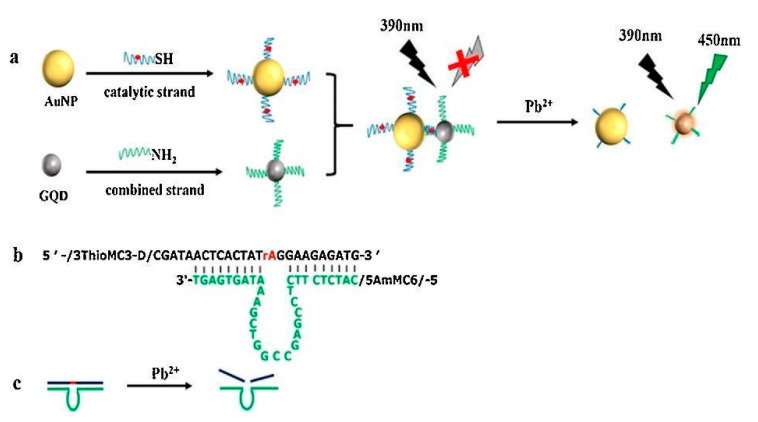
(**a**) Schematic illustration of Pb(II) detection based on energy transfer between graphene quantum dots (GQDs) and nanoparticles (NPs). (**b**) Structure formula of combined GQDs and AuNPs. (**c**) Scheme of catalytically cleave of the DNA molecule (reprinted from [100] with permission from Elsevier).

Another type of Pb(II) ions sensor has been proposed by Niu et al. [100]. In this case, they proposed a “turn-on” fluorescence sensor for Pb(II) detection based on graphene quantum dots and gold nanoparticles. Detection is based on FRET between GQDs and AuNPs. The detection mechanism is due to the fluorescence recovery from the separation of GQDs and AuNPs induced by adding Pb(II) as illustrated in Figure 6. GQDs were immobilized with amine modified combined strand by 1-Ethyl-3-(3-dimethyl-aminopropyl) carbodiimide (EDC). Then the AuNPs were modified with catalytic strand by sulfhydryl groups. GQDs and AuNPs are connected by catalytic strand (DNAzyme) and combined strand, which was single oligopeptide and modified on GQDs and AuNPs. The sensing system was highly sensitive and selective (evaluated against 11 metal ions) for the determination of Pb(II) with a LOD of 16.7 nM and a detection range from 50 nM to 4 µM.

An alternative method for Pb(II) ions detection based on the suppression of the surface energy transfer between acridine orange (AO) and AuNPs has been reported by Wang et al. [101]. In this case, the enhancement of fluorescence intensity is related to the concentration of Pb(II) in the 44 nM–4.8 μM range, with a LOD of 13 nM. The authors suggest that the interaction of AO with AuNPs causes a fluorescence quenching due to the gold nanoparticles surface energy transfer (SET). Pb(II) ions can also bind to AuNPs thanks to their strong aurophilic interactions, that can occupy the partial binding sites of AO on AuNPs along with the increase of free AO in the solution. As a result, the fluorescence of the system increases with the Pb(II) concentration. The system shows a good selectivity to lead ions in the nanomolar range, moreover this nanosensor is responsive to Mg(II) and Ca(II) but in very high concentrations range (millimolar). These interferences can be eliminated in real water samples by boiling and filtering the samples before the real analysis.

Another method was reported by Zhang et al. [102]. In this case the authors describe the synthesis of a fluorescent nanoprobe for sensitive fluorometric “turn-on” detection of Pb(II) based on AIE of the Au(I)-glutathione (GSH) complex. When Pb(II) ions are present in solution, the interaction with GSH causes the Au(I)-SG complexes to come in close proximity and as a consequence a strong fluorescence can be detected. This behavior can be used to design a method to quantify the amount of Pb(II) in solution. The sensitivity is strongly enhanced in the presence of ethanol due to the formation of dense Au(I)-SG nanoparticles. This system presents a good response in the range 2.0 to 350 μM and has a limit of detection of 0.1 μM together with a satisfactory selectivity.

A totally different approach was developed very recently by Singh et al. [103]. They employed an optical technique combining Rayleigh scattering of UV light (365 nm) and post-sample fluorescence detection from colloidal Ag nanoparticles having an SPR band at 420 nm. The light scattering from the AgNPs solution contaminated with Pb(II) ions saturate the light sensor even for very low concentration (ppm-order). It has to be noted that the pollutant is not detected at low concentrations at this wavelength. Instead, the fluorescence of a high-pass filter (cut off at 400 nm) at 520 nm was applied to detect pollutant in water in the high concentration range (up to several hundreds of ppm). The selectivity of the presented system is not described but it is referenced to another paper [104] where it is reported to be very high.

The Table 3 summarizes some of the most recently developed systems based on Au and Ag nanostructures used for heavy metal ions detection. In the table the sensing mechanism, the type of water pollutant and the LOD for each nano-system are reported.

## 4. Recent Developments on Pesticides Detection

The need to control insects, weeds and diseases has made pesticides a part of global agricultural production for millennia. The US Environmental Protection Agency (EPA) defines the term “pesticide” as “any substance or mixture of substances intended for (1) preventing, destroying, repelling or mitigating any pest, (2) use as a plant regulator, defoliant or desiccant or (3) use as a nitrogen stabilizer” [105]. Many pesticides persist in the environment and accumulate in the food chain, as occurs with organochlorine compounds such as dichloro-diphenyl-trichloroethane (DDT), the best known and historically the first pesticide to be used worldwide, which has been recognized to be a serious threat for the global health.

As such, the demand for highly sensitive pesticide sensors is pressing in order to control food safety, protect ecosystem and prevent disease. Accurate detection systems of these contaminants are available as chemical sensors, biosensors and electronical sensors [106,107]. However, besides high sensitivity and selectivity offered by those systems, the need for easy manipulation, rapid detection, disposability makes optical sensors for pesticides a good solution for on-field analysis and screening. In this section, we review some recent advances and new trends in optical sensors based on metal NPs for the detection of pesticide.

Generally, the sensor is constituted by an optical transducer component, which indicates the binding event of a target pesticide on a specific recognition unit (host-guest recognizer, molecularly imprinted polymers, antibody, aptamer and enzyme). The optical transduction can operate on the fluorescence intensity (fluorimetric) or on the absorption band position (colorimetric).

The great variety of pesticide sensors can be represented in three groups according to the different contaminant response mechanism: by direct modulation of the metal NP effect on the emission of some fluorophore; by the mediation of aptamers produced to specifically interact with particular pesticides; by the use of various biological molecules, (antibodies, nucleic acids, amino acids) directly conjugated with AuNPs which interact with the contaminants.

A simple biosensor based on AuNPs has been proposed for detection of organophosphorus (OP) compounds, including diazinon, iprobenfos and edifenphos [108]. The addition of imidazole to AuNPs suspended in water which contains OP compounds, causes the aggregation of AuNPs and changes the color of the suspension to purple. Indeed, a new peak at 660–670 nm appears within approximately 30 s. That constitutes a straightforward method for multiple OP sensing at ppb concentrations. In a similar way, a colorimetric sensor for the pesticide pymetrozine (PYM) was developed which measures the analyte-induced aggregation of melamine-modified gold nanoparticles. The system has a detection limit of 10 nM in a standard UV-Vis spectroscopic set-up [109].

Many transducer components operate by modulating the quenching role of metal NPs with respect to fluorophores active in the spectral region of the Au [61,63,110,111] or Au-Ag plasmon resonance [112]. A fluorescent sensor for the detection of thiodicarb has been constructed using Rhodamine B (RB) functionalized AuNPs. Following the adsorption of RB molecules on the surface of AuNPs, a fluorescence quenching occurs via FRET. The addition of thiodicarb to RB-AuNPs produced the recovery of the fluorescence since thiodicarb competitively adsorbs on the surface of AuNPs releasing RB molecules with restored radiative emission efficiency [35].

B,N-doped carbon quantum dots (CQDs) have been used to detect carbaryl pesticide. The sensing mechanism was based on the quenching of the 490 nm CQDs-fluorescence induced by AuNPs by inner filter effect. Such quenching can be suppressed and CQDs fluorescence recovered using thiocholine (TC) which induces the aggregation of AuNPs and, consequently, a drop in the absorption at 520 nm responsible of the inner filter effect. The specific action of carbaryl is to inhibit the acetylcholinesterase activity which catalyzes the hydrolysis of acetylthiocholine to TC. Therefore, a double signal assay method for the detection of carbaryl was established by measuring the fluorescence of CQDs and the absorbance of AuNPs [110].

**Figure 7:**
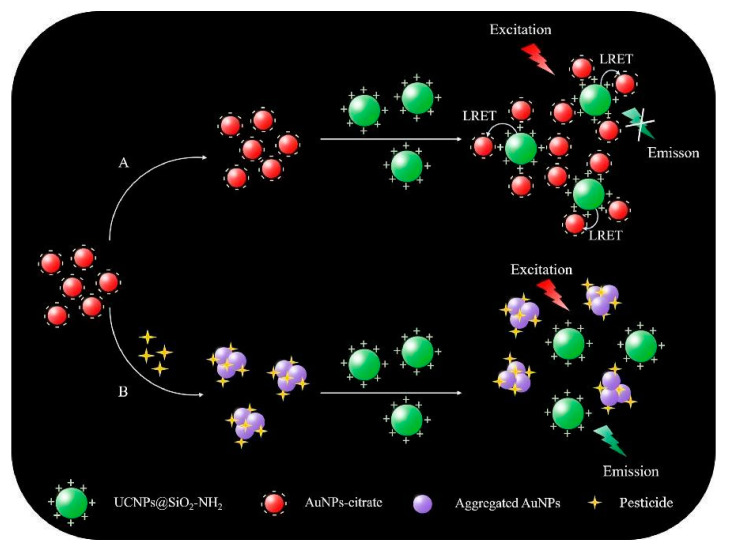
Schematic interpretation of the FRET method for the detection of cyano-containing pesticides. (Reprinted from reference [111] with permission from Elsevier).

Similarly, the fluorescence resonance energy transfer between up-conversion nanoparticles (UCNPs) and gold nanoparticles has been used to detect cyano-containing pesticides in herbal medicines (see Figure 7) [111]. Inhibitors of acetylcholinesterase (AChE) activity have been detected by a biosensing platform based on the FRET between boron nitride quantum dots (BNQDs) and AuNPs produced by the reduction of chloroauric acid into AuNPs by thiocholine from hydrolyzed acetylthiocholine (ATCh). The fluorescence quenching of BNQDs was weakened by an organophosphorus pesticide, paraoxon, used to lower the activity of AChE and hinder the enzymatic hydrolysis reaction [61].

**Figure 8:**
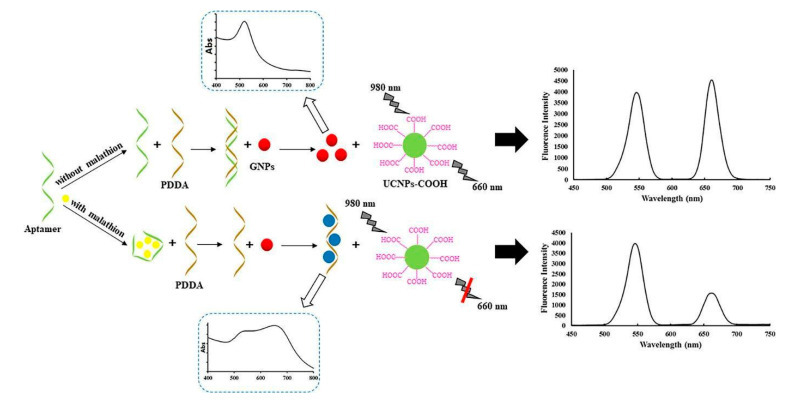
Schematic illustration of the aptasensor for malathion detection based on FRET between NaYF_4_:Yb,Er and gold nanoparticles (GNPs). (Reprinted from reference [62] with permission from Elsevier).

The use of aptamers that can be chosen to bind specifically to some molecules, allows the development of optosensors highly selective to a particular target pesticide. Specific aptamers have been used to monitor malathion contents in adulterated tap water [62]. The aptamers bind to cationic polymer through electrostatic interactions. However, the capture of the organophosphorus pesticide hinders such electrostatic bond to the cationic polymer leaving the latter free to encapsulate gold nanoparticles which interact efficiently with negatively charged up-conversion fluorescent nanoparticles (UCNPs) producing fluorescence resonance energy transfer (see Figure 8). Therefore, the combination results in UCNP fluorescence quenching and the degree of quenching is correlated with the concentration of malathion. Aptamer were also used to modify up-conversion nanoparticles (Apt-UCNPs) that were conjugated with graphene oxide (GO) through π-π interaction. Due to the FRET between UCNPs and GO, the fluorescence is quenched. However, the aptamer preferentially binds with diazinon. Therefore the addition of OP pesticides causes the separation of GO and, consequently, the enhancement of fluorescent signal [113].

The proposed nanosensor showed an efficient, specific and simple approach for the detection of diazinon in food, with a high potential for food safety and quality control.

A fluorescent aptasensor in aqueous solution was proposed for the detection of carbendazim (CBZ), a widely used, systemic, broad-spectrum benzimidazole fungicide; it used CBZ-specific aptamer as sensing probe and AuNPs/RB as indicator, respectively [114]. The naked aptamer wraps AuNPs and maintains the nanoparticles dispersed in NaCl solution. On the other hand, when the aptamer specifically combines with CBZ it forms a stable complex that leaves AuNPs exposed to the aggregating effect of the NaCl solution. The dispersed AuNPs efficiently quench the RB fluorescence, while after aggregation loose the capability to impair the fluorescent indicator.

A colorimetric and fluorometric method using AuNPs, up-conversion nanoparticles (UCNPs) and an acetamiprid-binding aptamer (ABA) has been proposed for ultrasensitive and selective detection of the neonicotinoid pesticide acetamiprid [115]. The ABA is configured into a duplex with a complementary DNA covalently attached to AuNPs. The resulting double-stranded DNA-functionalized AuNP probe aggregates in 0.15 M NaCl solution. In the presence of acetamiprid, the ABA undergoes a structural switch from a DNA duplex to an aptamer-acetamiprid complex and consequently dissociates from the AuNPs that again turn stable against salt-induced aggregation. Acetamiprid in the 0.025–10 μM concentration range can tune the ratio of AuNP probe absorbance at 524 nm (red) and 650 nm (purple blue). The colorimetric method is accompanied by a fluorimetric detection obtained by the introduction of DNA-modified carboxylated UCNPs (silica-coated NaYF4:Yb,Er) which display red and green fluorescence under 980 nm excitation. An inner filter effect occurs between DNA-modified UCNPs and double-stranded DNA-modified AuNPs. A 0.36 nM detection limit was reached by this coupled technique.

Alternatively, the inner filter effect of AuNPs with respect to fluorescence of carbon dots has been modulated by S-18 aptamer depending on the presence of acetamiprid [73].

Various biological molecules, such as antibodies [116,117], nucleic acids [118,119,120], amino acids [121,122] can be used for direct conjugation with AuNPs. Fluorescence-based immune assay techniques have been used to detect different type of pesticides [123,124,125]. Zhang et al. [123] used fluorescently labelled oligonucleotide and AuNP signal amplification technology to develop a multi-analyte fluorescence immunoassay for detection of three organophosphate pesticides (triazophos, parathion and chlorpyrifos) in various agro-products (rice, wheat, cucumber, cabbage and apple).

**Figure 9:**
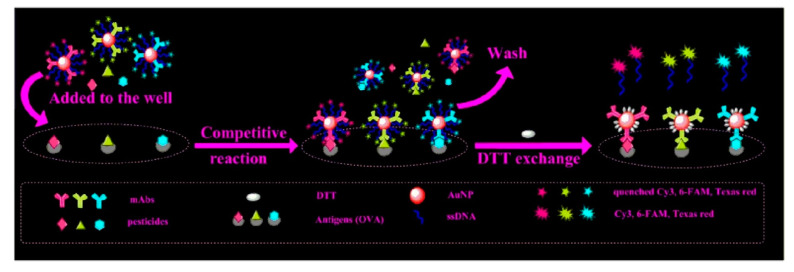
Scheme of the fluorescence immunoassay based on oligonucleotide signal amplification. (Reprinted from reference [123] with permission of Elsevier).

The simultaneous modification of the corresponding antibodies and fluorescently labelled oligonucleotides on the probe surface achieved the goal to realize a multi-labelled AuNP probes for the three tested pesticides (see Figure 9). The three selected fluorophores (6-FAM, Cy3 and Texas red) show high fluorescence intensity and little overlap of excitation/emission wavelengths. The AuNP probes for the three analytes were prepared by simultaneously modifying the corresponding oligonucleotides (fluorescent labelled) and monoclonal antibodies (mAbs) on the surface of colloidal gold.

Table 4 summarizes some of the most recently developed systems used for pesticides detection. The limit of detection, the kind of pesticide and the detection technique involved for each nanosensors are reported. The nanostructures are also subdivided according to the three different contaminant response mechanism.

## 5. Conclusions

Fluorescent probes based on noble metal nanomaterials represent a multi-versatile detection tool that has excellent sensitivity for the detection of a wide range of pesticides or heavy metal ions. These capabilities combined with other advantages, such as faster detection times, simpler protocols, in situ sampling, portability and reduced cost, promote these materials as valid alternatives with respect to the most common analytical techniques.

We have reviewed the most recent achievements in the field of metal nanostructures for environmental pollutant detection based on fluorescence. We have discussed the most common photo-physical effects acting on the fluorescence of the noble metal nanomaterials and the effects induced by the presence of pollutant species. More in detail, we have described photoinduced electron transfer and photoinduced charge transfer processes, fluorescence resonance energy transfer mechanism, aggregation-induced emission effect and the inner-filter effect. In conclusion, we have presented a list of recent and promising systems used for detection of heavy metal ions and pesticides, highlighting the performances in terms of sensitivity, linear range, selectivity and limit of detection.

## Figures and Tables

**Table 3:** List of noble metal nanomaterials used for heavy metal ions detection and their limit of detection (LOD).

Sensing mechanism	Analyte	LOD	Ref.
Thiol-DNA-functionalized gold nanoparticles (AuNPs) quenched by a formation of a complex induced by Ag(I)	Ag(I)	9.5 nM	[33]
Glc-CuNPs quenched by aggregation induced by Ag(I)	Ag(I)	100 µM	[77]
SiNPs quenched by aggregation induced by Ag(I)	Ag(I)	0.457 µM	[78]
	Hg(II)	2.676 µM	
AuNCs covered with glutathione enhanced the luminescence due to aggregation-induced emission of the functionalized gold nanoclusters by LMCT or LMMCT	Ag(I)	0.2 nM	[79]
AgNCs-dBSA quenched by Hg-Ag metallophilic interaction	Hg(II)	10 nM	[90]
AgNCs-DHLA quenched by aggregation induced by Hg(II)	Hg(II)	10^−10^ M	[91]
AuNCs quenched by Hg-Au metallophilic interaction	Hg(II)	0.5 nM	[92]
AuNCs-Ag@Keratin quenched by Hg-Au and Hg-Ag metallophilic interactions	Hg(II)	2.31 nM	[93]
AuNPs-MUA aggregation-induced fluorescence quenching by Hg(II)	Hg(II)	5 nM	[34]
AuNPs–MSD-FAM sensor based on NSET	Hg(II)	16 nM	[94]
AuNPs-DNA-FAM sensor based on FRET	Hg(II)	8 nM	[65]
AuNPs-QDs sensor based on time-gated FRET	Hg(II)	0.87 nM	[64]
AuNPs-RB-HDT nanocomposites system, the FRET effect from RB to AuNPs, Hg(II) ions induce AuNPs aggregation as a consequence the RB is not quenched anymore	Hg(II)	0.5 ppb	[95]
AuNCs -PCL nanofibers quenched by Au-Hg amalgam formation	Hg(II)	50 ppt	[96]
SiNPs sensor based on IFE	Cr(VI)	28 n M	[80]
AuNCs-BSA and CDs, IFE mechanism for Cr(VI) and Au-Hg interaction for Hg(II)	Cr(VI) Hg(II)	5.34 nM 1.85 nM	[81]
AuNCs-GSH Cr(III) ions may induce aggregation or superficial imperfections of NCs	Cr(III)	2.5 µg/L	[82]
AuNCs-GSH redox interaction between Cr(VI) and GSH	Cr(VI)	0.5 µg/L	[82]
Orange-emitting fluorescence AuNCs with methitione sensor based on AIE	Cd(II)	12.25 nM	[83]
AuNCs-MUA-CE sensor based on CHEF or AIE in cases of Cd(II) and Zn(II), while Cu(II) ions induce a not fluorescent aggregation of NCs	Cd(II) Zn(II) Cu(II)	12 nM 16 nM 26 nM	[67]
AuNCs-BSA, Co(II) ions induce intersystem crossing, while Cd(II) induce FRET on the system	Cd(II) Co(II)	- -	[84]
AuNCs-CQDs ratiometric-based sensor, Cd(II) induces an electrostatic interaction with AgNCs ligands, quenching the PL emission only of the AgNCs	Cd(II)	105 nM	[74]
OMRTH-AgNPs, Cd(II) induces aggregation of NPs	Cd(II)	10^−8^ M	[85]
R-PE-AgNPs, Cu(II) induces aggregation of NPs	Cu(II)	19 nM	[87]
AuNCs-BSA, Cu(II) ions reversible interact with BSA ligands, while Hg(II) ions irreversible interact with Au on the NCs surface	Cu(II) Hg(II)	300 nM 8 nM	[89]
SiNPs, Mn(II) induces aggregation of NPs	Mn(II)	1.1 µM	[97]
CuNCs-Thymine, Mn(II) induces AIE on the system	Mn(II)	10 µM	[98]
DNA-AgNCs, Pb(II) ions interact with DNA strands, giving rise to more stable molecular conformation, PL emission is enhanced	Pb(II)	0.3 nM	[99]
GQDs-AuNPs, Pb(II) ions induce the separation of GQDs and AuNPS, restoring the GQDs emission	Pb(II)	16.7 nM	[100]
AuNPs-AO, Pb(II) ions inhibit the SET between AO and AuNPs	Pb(II)	44 nM	[101]
Au(I)-SG complexes, Pb(II) ions promote the AIE on Au(I) complexes	Pb(II)	0.1 µM	[102]

**Table 4:** List of metal nanomaterials for pesticides detection with the interaction mechanisms and the LOD values.

Detection Technique	Analyte	LOD	Ref.
**Direct Metal-NP**			
FRET between BNQDs and AuNPs	Paraoxon	33 ng/L	[61]
NSET of AuNPs on the fluorescence of fluorescein	Fenitrothion	9.41 nM	[63]
Recovery of luminescence of B,N-doped CQDs quenched by Au-NP + thiocoline	Carbaryl	0.06 µg/L	[110]
Modulation of luminescence resonance energy transfer (LRET) between UCNPs and AuNPs	Acetamiprid	15 ng/L	[111]
	Fenpropathrin	240 ng/L	
	Chlorothalonil	11 ng/L	
RB-Ag/AuNPs turn-on effect due to interaction between pesticide and RB-Ag/AuNPs	Parathion methyl	1.8 pg/mL	[112]
Competitive adsorption between RB and thiodicarb on the surface of AuNPs	Thiodicarb	0.08 ppm	[35]
**Aptasensors**			
Negatively charged UCNPs and cationic-polymer encapsulated GNPs exploit the FRET mechanism between malathion and UCNPs-GNPs	Malathion	1.42 nM	[62]
Fluorescent aptasensor by modulation of IFE on CDs fluorescence	Acetamiprid	1.08 μg/L	[73]
Aptamer-modified UCNPs based on FRET	Diazinon	23 ng/L	[113]
Fluorescence quenching of specific aptamer by AuNPs	Carbendazim	2.33 nM	[114]
Colorimetric method with Acetamiprid-binding aptamer functionalized with AuNPs	Acetamiprid	0.36 nM	[115]
**Antibodies, nucleic acids, amino acids**			
Adenosine Triphosphate-Modified AuNPs by colorimetric method	Ethoprophos	37.0 nM	[118]
Colorimetric aptasensors based on DNA and AuNPs	Tobramycin	23.3 nM	[119]
Fluorescent polarization immunoassay using a broad-specificity monoclonal antibody	Organophosphate pesticides	10 ng/mL	[120]
Fluorescence quenching-based recombinant fluorescent-peptide tracer	Imidaclothiz	8.00 ng/mL (sensitivity)	[122]
AuNP functionalized with fluorescently labelled oligonucleotide	Triazophos	0.007 μg/L	[123]
	Patathion	0.009 μg/L	
	Chlorpyrifos	0.087 μg/L	
Immunochromatographic strip	Fenitrothion-IgG	5 mg/mL	[124]
Fluorescence-based immunoassay based on the quenching effects of functionalized AuNCs	Imidacloprid	1.3 ng/mL (IC_50_)	[125]

## Data Availability

No new data were created or analyzed in this study. Data sharing is not applicable to this article.
